# Modelling Hydrology of a Single Bioretention System with HYDRUS-1D

**DOI:** 10.1155/2014/521047

**Published:** 2014-07-15

**Authors:** Yingying Meng, Huixiao Wang, Jiangang Chen, Shuhan Zhang

**Affiliations:** ^1^College of Water Sciences, Beijing Normal University, Beijing 100875, China; ^2^Beijing Water Science and Technology Institute, Beijing 100048, China

## Abstract

A study was carried out on the effectiveness of bioretention systems to abate stormwater using computer simulation. The hydrologic performance was simulated for two bioretention cells using HYDRUS-1D, and the simulation results were verified by field data of nearly four years. Using the validated model, the optimization of design parameters of rainfall return period, filter media depth and type, and surface area was discussed. And the annual hydrologic performance of bioretention systems was further analyzed under the optimized parameters. The study reveals that bioretention systems with underdrains and impervious boundaries do have some detention capability, while their total water retention capability is extremely limited. Better detention capability is noted for smaller rainfall events, deeper filter media, and design storms with a return period smaller than 2 years, and a cost-effective filter media depth is recommended in bioretention design. Better hydrologic effectiveness is achieved with a higher hydraulic conductivity and ratio of the bioretention surface area to the catchment area, and filter media whose conductivity is between the conductivity of loamy sand and sandy loam, and a surface area of 10% of the catchment area is recommended. In the long-term simulation, both infiltration volume and evapotranspiration are critical for the total rainfall treatment in bioretention systems.

## 1. Introduction

Rapid urbanization in watershed, with the increasing impervious area, implies both larger stormwater runoff volumes and peak flows and consequently reduces other components of the hydrologic cycle, for example, infiltration and evapotranspiration. Moreover, stormwater directly transports harmful substances from urban surfaces to downstream water systems, thus degrading the water quality. The negative impacts of urban stormwater have received widespread recognition [1], and maintaining stormwater quantity (e.g., flood peak and total volume) and quality (e.g., pollution) as close as the predevelopment levels has become increasingly popular. Bioretention, also known as rain garden, biofilter, or biofiltration, is a terrestrial-based water quantity and quality control practice that can be designed to mimic predevelopment hydrology (PGCo, 2007). It is thus commonly used as a source control technique to manage stormwater runoff in areas under urbanization and a retrofit technique in already developed areas [2]. Bioretention has also played an important role in the implementation of best management practice (BMP) and low impact development (LID) in America, water sensitive urban design (WSUD) in Australia, and sustainable urban drainage system (SUDS) in England.

There are many factors influencing the performance of bioretention systems, such as type of vegetation, depth of the filter media, size of the system relative to its catchment, and type of soil. Sizing, vegetation, construction technique, and soil mixture were all reported to have an important influence on the hydraulic conductivity of bioretention [3, 4]. The sizing of biofilters was also emphasized by Brown and Hunt III [5] who presented better reductions in runoff volume with deeper media depth. Furthermore, the hydraulic conductivity of the underlying soil and the internal water storage zone depth were also considered as primary factors influencing water reduction [6]. Overall, this research work on factors influencing the performance of bioretention was mainly based on column studies in laboratories or field studies [4, 5]. Because experimental observations were easily restricted by test conditions, unexpected results were sometimes reached. In the field study of Brown and Hunt III [5], for example, the surface storage volume of two bioretention cells was undersized because of design and construction errors, having substantial negative impacts on cell performance. Therefore, there is an increasing need to predict the hydrologic and water quality performance of bioretention systems using hydrologic model, which could be conveniently used in design, evaluation, or other purposes.

Initial model studies about bioretention did not include underdrains; for example, Heasom et al. [7] have attempted to predict the overflow volume in a bioinfiltration cell using one-dimensional hydrological model HEC-HMS. Considering an underdrain, He and Davis [8] developed a two-dimensional model simulating the subsurface flow. However, both models were based on individual rainfalls and were unable to perform continuous simulations and therefore they could not account for the changes in soil moisture conditions from previous rainfall events. The RECARGA model [9], widely used in the design and performance assessment of bioretention systems [10], allows for both continuous modelling and single-event modelling, but its minimum hourly rainfall interval makes it unable to conduct simulations for very short periods. Moreover, some parameters such as the number of underdrains and their depths and types of filter media could not be specified by the user, limiting the model's applications in some situations. As the water movement process in bioretention cells installed with underdrains is very similar to agricultural drainage pipes, modelling hydrologic performance in bioretention systems with DRAINMOD, an agricultural drainage model, has been common in recent years [11]. But DRANIMOD is unsuitable for conducting short-term simulations with a minimum calculation time of 1 month. Other models used in bioretention simulations involve SWMM (USEPA, 2010), SUSTAIN (USEPA, 2013), or MUSIC (eWater, 2013), but because of scale problem they are not appropriate for a single facility simulation.

One potential solution to reduce the frequent urban waterlogging disasters in Beijing in recent years is to control urban runoff at source as much as possible. As a source control technique, bioretention systems have advantages in ultraurban areas such as Beijing where land is unavailable for large control practices such as retention ponds, grassed swales, and constructed wetlands. The main objective of this study is to evaluate the hydrologic performance of bioretention facilities and provide instructive guidance for their design and application in Beijing. We developed a model tool to predict the hydrologic performance of a single bioretention facility and discuss the influence of different design parameters. Overall, the above-mentioned models have their own shortcomings in terms of scale, calculation time, configuration design, and other aspects. Comparatively, the HYDRUS-1D model [12] is a more appropriate model, with flexible water flow boundary conditions, a minimum calculation interval of 1 s, and unlimited simulation time. Hilten et al. [13] and Ladu et al. [14] both simulated the stormwater performance of a green roof using HYDRUS-1D. As a heterogeneous multilayer soil medium such as the green roof system, a bioretention system has the potential to be simulated by this model, while no data have yet been reported in the literature on the model's ability to model bioretention performance. In our study, the HYDRUS-1D model was devised based on input variables measured at two bioretention cells constructed in Beijing. The hydrology processes were measured at these facilities for nearly 4 years, and the collected data were used to calibrate and validate the model. The factors affecting the bioretention performance are discussed based on the simulation results for different design storms, filter media depths and types, and surface areas.

## 2. Methods

### 2.1. Principle of the HYDRUS-1D Model

The hydrologic processes in bioretention systems consist of evapotranspiration, infiltration, and runoff generation. The water balance equation is given as follows:

(1) $$\begin{array}{c} \text{ET}=P-I-R\pm \text{DSW}, \end{array}$$

where ET is the evapotranspiration,* P* is the precipitation,* I* is the infiltration,* R* is the runoff, and DSW is the change in soil water content. The total runoff *R* is given as follows:

(2) $$\begin{array}{c} R=\text{RS}+\text{RB}, \end{array}$$

where RS is the surface runoff and RB is the bottom runoff from the drainage layer.

### 2.2. Field Study Site

**Figure 1:**
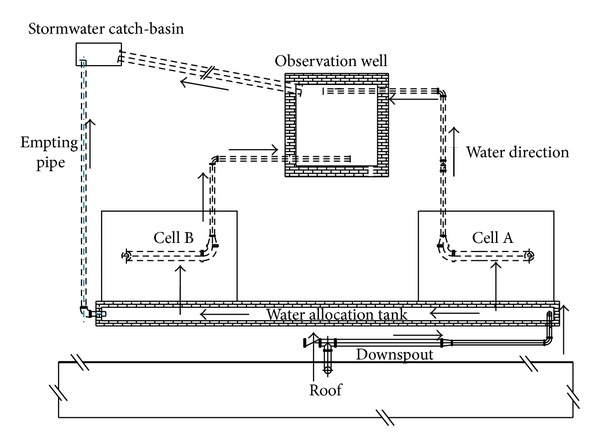
Plan of the bioretention study area (not to scale).

**Figure 2:**
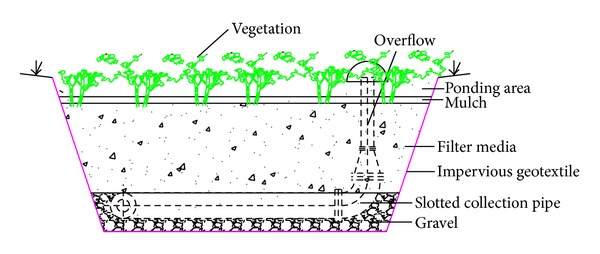
Schematic view of the bioretention cell.

Two parallel bioretention cells (Cells A and B) were constructed in Mentougou district in Beijing in 2010 (Figure 1). Each cell was 3 m ∗ 2 m on the top surface, 2.2 m ∗ 1.1 m on the bottom surface, and 1.1 m deep. Being designed to capture and treat stormwater runoff from a 60 m^2^ impervious roof, each cell covers 10% of the catchment area. The cells were built with the following composition (Figure 2):

- a drainage layer at the base, containing a 110 mm diameter slotted PVC pipe (connected to the observation well for flow measurement) surrounded by 30 mm gravel with the diameter of 5–10 mm;
- sixty centimeters of filter media: conventional media in Cell A, 97% of sand and soil, 3% of peat (both by volume); two layers in Cell B, one-third of sand and soil and two-thirds of blast furnace slag with the diameter of 5 mm on the top layer of 25 cm (both by volume), vermiculite on the bottom layer of 35 cm with the diameter of 0.5–5 mm to increase soil porosity [4];
- five centimeters of mulch: shredded pine bark;
- vegetation cover, with native plants of* Ophiopogon japonicas* and* Iris tectorum* for Cells A and B. Before choosing vegetation to conduct experiments on, we defined some criteria to ensure the vegetation suitable for bioretention construction. The criteria were defined to fit the climate in Beijing, specifically, drought, flood, pollution, salt, shade, and cold tolerant, with ornamental value locally selected, low cost, and low maintenance. Based on these requirements, we finally chose* Ophiopogon japonicas* and* Iris tectorum*;
- an overflow drain connected to the PVC pipe allowing a maximum ponding depth of 15 cm;
- an impervious geotextile on the sides and bottom to minimize migration of water into or out of the system.

### 2.3. Field Measurements

The infiltration and overflow volume which contributed to the bottom runoff both discharged through the PVC pipe, and its flow rate was measured using a 5 L measuring cup and a stopwatch every 5 min after the PVC pipe started to drain off water in the observation well, until there is no pipe flow. If ponding occurred, the water level in the ponding area was measured by a meter ruler every 5 min until ponding disappeared. These in situ data were collected during June to September in 2010, 2011, 2012, and 2013.

In the absence of natural rainfall, simulated stormwater was sometimes used for the experiments, using the reference method provided in Hsieh and Davis [15] to prepare the simulated stormwater. In artificial rainfall, the simulated stormwater with the theoretical volume from each cell's catchment area was mixed well and pumped into the cells evenly over an hour. The bottom runoff and ponding processes were also monitored.

Thirty-eight rainfall events were monitored, with thirty-three artificial events and five natural events. The artificial rainfall test results were mainly used for parameter calibration and validation of the HYDRUS-1D model, and the natural rainfall results were all used for model validation.

### 2.4. Modelling with HYDRUS-1D

Input requirements for HYDRUS-1D include geometry and time information, soil hydraulic and vegetation properties, initial and boundary conditions, and meteorological information, whose values are listed in Table 1. The soil hydraulic parameters used in the van Genuchten model were measured using a high-speed centrifuge method (Table 2). Daily measurements of meteorological variables, including air temperature and humidity, atmospheric pressure, precipitation, wind speed and direction, and incoming shortwave and longwave radiation, were collected from the meteorological station in Beijing (number 54511) near the experimental site. The initial conditions are given in terms of water content, which is linearly distributed in the soil profile. The other parameters were specified according to the default values.

In this study, the model was validated based on the measured bottom runoff and water level. Because the artificial and natural rainfalls were both individual events, we chose an artificial rainfall and a natural rainfall, respectively, as the rainfall events for validation. Statistics of the root-mean-square error (*RMSE*), mean relative error (*MRE*), and correlation coefficient (*R* ^2^) were then used to assess the accuracy of the simulation. The smaller the* RMSE* and* MRE *are, the more closely the *R* ^2^ approximated to zero and the better the model performed.

### 2.5. Model Application

Upon verifying the accuracy of the HYDRUS-1D model, simulations were run using different input variables to optimize the design of the bioretention systems. Four general scenarios with different rainfall return periods, filter media depths and types, and surface areas were investigated, and, through scenario analysis using the model, the influence of the different design parameters on the hydrologic performance of bioretention systems was studied. In the above simulations, storms were simulated as independent events. As HYDRUS-1D had no limitation on simulation time, the long-term hydrologic performance of bioretention systems was also assessed by inputting the annual meteorological data in 2012.

The hydrologic performance of bioretention systems could be described in terms of the hydrologic effectiveness and the water detention and retention effects. Hydrologic effectiveness (*R*_hydro_) denotes the total rainfall runoff treated by the bioretention in whatever form, specified using (3). Water detention means water temporarily reserved by the system, which was demonstrated by bottom runoff delay (Δ*t*_dd_), bottom runoff peak flow delay (Δ*t*_pd_), and bottom runoff peak flow reduction (*R*_pr_), given in (4)–(6). Comparatively, water retention (*R*_reten_) refers to the water completely reserved by the system, given by (7). The ponding duration (*t*_pond_) can also reflect the hydrologic performance to some extent. Consider

(3) $$\begin{array}{c} R_{\text{hydro}}=\frac{V_{\text{inflow}}}{V_{\text{runoff}}}\times 100\%, \end{array}$$

where *V*_inflow_ is the inflow volume of the bioretention cell and *V*_runoff_ is the runoff volume from the catchment area. Consider

(4) $$\begin{array}{c} \Delta t_{\text{dd}}=t_{\text{drain}}-t_{\text{inflow}}, \end{array}$$

where *t*_drain_ is the time the bottom runoff appears and *t*_inflow_ is the time the inflow enters the bioretention cell. Consider

(5) $$\begin{array}{c} \Delta t_{\text{pd}}=t_{p\text{drain}}-t_{p\text{inflow}}, \end{array}$$

where *t*_*p*drain_ is the time the bottom runoff peak appears and *t*_*p*inflow_ is the time the inflow peak appears. Consider

(6) $$\begin{array}{c} R_{\text{pr}}=\frac{q_{p\text{inflow}}-q_{p\text{drain}}}{q_{p\text{inflow}}}\times 100\%, \end{array}$$

where *q*_*p*inflow_ is the inflow peak and *q*_*p*drain_ is the bottom runoff peak. Consider

(7) $$\begin{array}{c} R_{\text{reten}}=\frac{V_{\text{inflow}}-V_{\text{drain}}}{V_{\text{runoff}}}\times 100\%, \end{array}$$

where *V*_drain_ is the bottom runoff volume.

## 3. Results and Discussion

### 3.1. Model Validation

**Figure 3:**
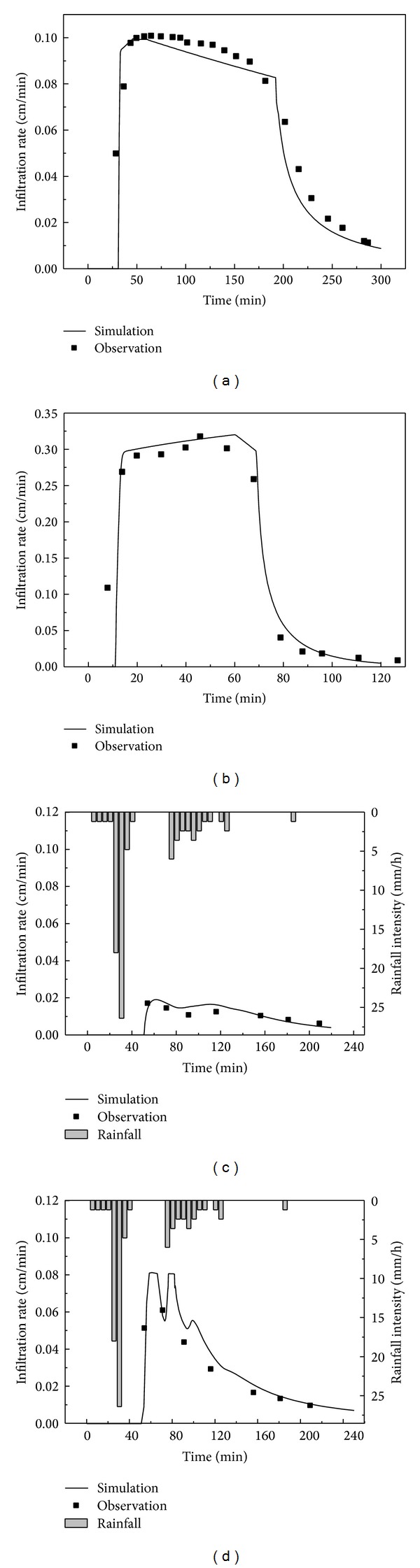
Observed and simulated infiltration rate in the artificial rainfall for validation in (a) Cell A and (b) Cell B and in the natural rainfall for validation in (c) Cell A and (d) Cell B.

In our experiments, overflow never occurred; thus, the bottom runoff volume was equal to the infiltration volume. Dividing the infiltration volume by the surface area of the bioretention cell, we obtain the infiltration rate. The comparison of the modelled infiltration rate by HYDRUS-1D and the observation values in Cells A and B are shown in Figure 3, with all of the simulated values in good agreement with observed ones. Because the filter media of Cell B had added blast furnace slag and vermiculite whose particle sizes were larger than soil particles, it was reasonable that the infiltration rate was higher in Cell B in both the artificial and the natural rainfalls, and the bottom runoff delay was obviously later in Cell A in the artificial rainfall.

The statistics for the* RMSE*,* MRE*, and *R* ^2^ between the simulated and observed values are shown in Table 3. The evaluation results were acceptable with the* RMSEs* all approximating zero, the* MRE* almost at 0.20, a higher *R* ^2^ (>0.9) in the artificial rainfall, and a relatively smaller *R* ^2^ (>0.6) in the natural rainfall, which might be the result of fewer observed data.

**Figure 4:**
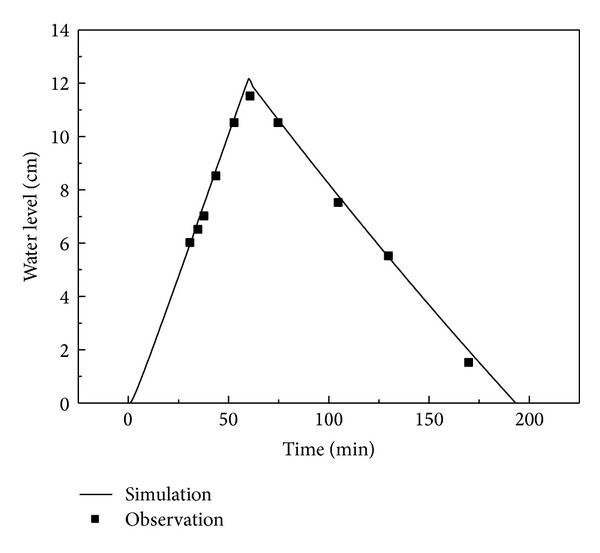
Observed and simulated water level in the artificial rainfall for validation in Cell A.

For the ponding process, the simulated and measured water levels in Cell A in the artificial rainfall for validation are presented in Figure 4. The observed and calculated values were well matched, as the* RMSE*,* MRE,* and *R* ^2^ were 0.34, 0.059, and 0.99, respectively. Flooding never occurred in Cell B in the experiments, while a thin layer of water (less than 2 cm) appeared in the simulation results, probably because the water retention of mulch is not taken into account by the model.

Overall, the HYDRUS-1D model was capable of capturing the hydrologic processes in bioretention systems with reasonable accuracy and can thus be used to assess bioretention performance under different circumstances.

### 3.2. Design Parameters Optimization

#### 3.2.1. Rainfall Return Periods

**Figure 5:**
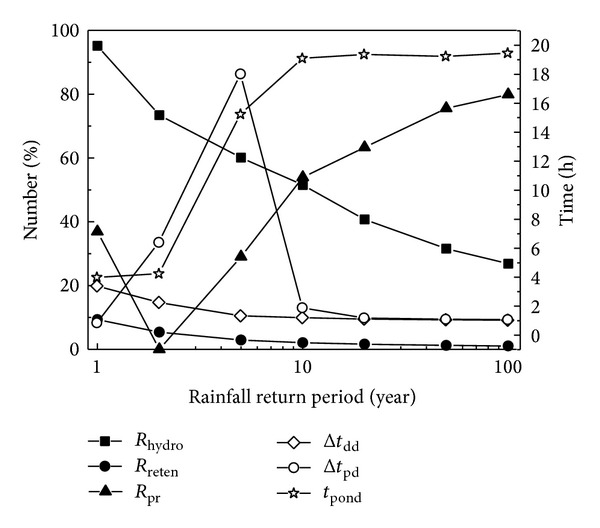
Hydrologic performance of bioretention measures under rainfalls with different return periods.

Twenty-four-hour design storms with different return periods in the central area of Beijing were calculated according to the* Hydrologic Manual of Beijing* [16]. With the other input requirements the same as those in the Cell A simulation, the simulated results for different design storms are presented in Figure 5. From Figure 5, it is clear that the hydrologic effectiveness (*R*_hydro_) decreased with the rainfall return period because of the increased total runoff volume from the catchment area. Thus, in rainfalls with return periods greater than 5 years, the peak flow reduction (*R*_pr_) was higher because *R*_hydro_ was lower, and only in rainfalls with return periods smaller than 2 years did *R*_pr_ really increase because of the higher *R*_hydro_. Therefore, greater hydrologic effectiveness is the premise for better water treatment. The water detention effect can be further evaluated using the bottom runoff delay (Δ*t*_dd_) and bottom runoff peak flow delay (Δ*t*_pd_) besides *R*_pr_, and Δ*t*_dd_ gradually decreased when the rainfall return period was greater than 5 years, while an obvious decrease was observed with a rainfall return period smaller than 5 years. However, Δ*t*_pd_ changed dramatically with the rainfall return period, with a sharp increase for a return period smaller than 5 years, a significant decrease for a return period between 5 and 10 years, and a gentle decrease for a return period greater than 10 years. This is because of the two peaks of the 24-hour design storm in Beijing, and the bioretention could only abate the first peak with a rainfall return period greater than 10 years, while, in rainfalls with a smaller return period, it successfully eliminated the first peak, resulting in the delayed bottom runoff peak caused by the second rainfall peak. Furthermore, the variation in ponding duration (*t*_pond_) was very similar to Δ*t*_dd_, with a gradual increase in rainfalls for a return period greater than 5 years, while there was a significant increase in rainfalls with a return period smaller than 5 years. It should be noted that to prevent mosquito breeding and maintain vegetation growth ponding duration is required to be shorter than 48 h (PGCo, 2007), and the simulated *t*_pond_ between 4 and 20 h under different design storms all met the requirement. In summary, the water detention effect in bioretention systems is much better in rainfalls with smaller return periods. Referring to the total water retention effect, the decrease in water retention (*R*_reten_) with the rainfall return period was not obvious, and *R*_reten_ was smaller than 10% even with the smallest storm return period of 1 year, showing that bioretention measures could store very limited stormwater, which might be because of the completely impervious sides and bottom surface in our study site.

Conclusively, with impervious surroundings, bioretention facilities only regulate the inflow runoff discharge process, their total water retention effect is very limited, and their detention effect is significantly better in small rainfalls with return periods smaller than 2 years. This agrees with the research results of Davis [17] and Li et al. [18], who indicated that the greatest impact was noted for the smaller events. Thus, a 2-year storm or less is recommended for use as the design storm for bioretention measures. Therefore, the following discussion on the design parameters is based on a 1-year storm.

#### 3.2.2. Filter Media Depths

**Figure 6:**
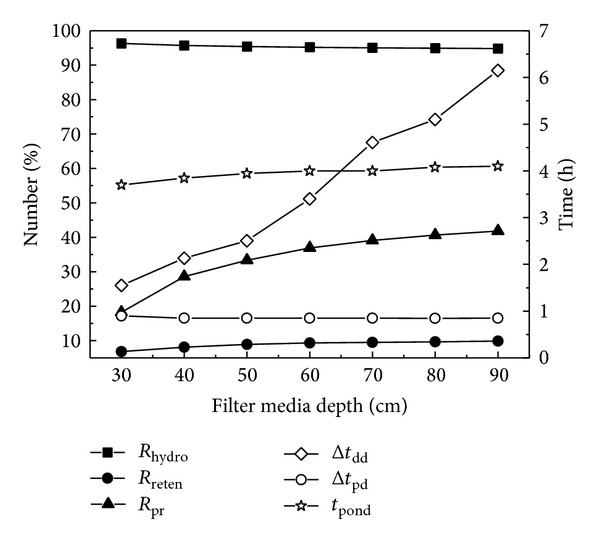
Hydrologic performance of bioretention measures under different filter media depths.

Taking into account the demand for water quality improvement [19] and the underdrains successfully connected with the municipal storm sewer, the input filter media depths were from 30 to 90 cm. The variation in hydrologic performance is presented in Figure 6. The bottom runoff peak flow reduction (*R*_pr_) and bottom runoff delay (Δ*t*_dd_) increased significantly with the filter media depth, while the total water treated (*R*_hydro_), water retention (*R*_reten_), bottom runoff peak flow delay (Δ*t*_pd_), and ponding duration (*t*_pond_) showed almost no change. This indicated that the filter media depth only has a beneficial impact on water detention, with better water detention in deeper filter media, while it has little or no influence on hydrologic effectiveness, water retention, and ponding control, which may also be attributed to the impervious surroundings. As in the study of Brown and Hunt III [5], the exfiltration volume in bioretention systems without the impermeable membrane is much higher in the deeper media cells because of greater storage volume in the media and more exposure to the side walls, leading to the better reductions in runoff volume.

As one of the main costs in constructing bioretention cells, the filter media depth should not be too great; thus, a cost-effective depth should be used in bioretention design. This could also refer to the media depth requirements in North Carolina, where vegetation is the determined factor and the minimum media depth is 0.6 m for cells vegetated with grass or shallow rooted plants and 0.9 m for cells vegetated with shrubs or trees (NCDENR, 2009).

#### 3.2.3. Types of Filter Media

**Figure 7:**
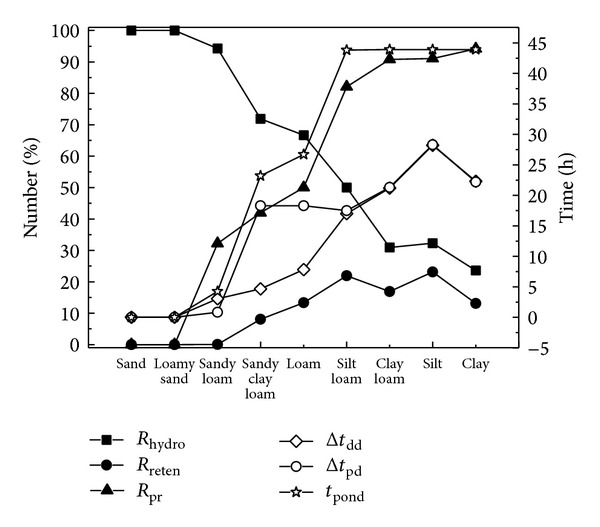
Hydrologic performance of bioretention measures under different filter soils.

Nine soils, from sand to clay with declining saturated hydraulic conductivity, included in the soil catalog of the HYDRUS-1D model, were used as input filter media, and in each case the hydrologic performance was simulated with default soil hydraulic parameters in the model (Figure 7). It was obvious that filter soil played an important role in hydrologic effectiveness and the total water treated by the system (*R*_hydro_) decreased with declining hydraulic conductivity from sand to clay. This agreed with the research results of Le Coustumer et al. [2] who even proposed that the initial specified hydraulic conductivity of filter media was the critical determinant of the long-term hydraulic behavior of a biofilter. In this study, with a high infiltration rate, the sandy texture soils treated all the rainfall runoff without ponding, while with low infiltration rate the clay soil only treated 23.6% of the total rainfall runoff while it overflowed the other runoff and was always ponding. Without higher hydrologic effectiveness, the obvious increase in the water detention parameters of bottom runoff peak reduction (*R*_pr_), bottom runoff delay (Δ*t*_dd_), and bottom runoff peak flow delay (Δ*t*_pd_) with the declining infiltration rate makes no sense. Moreover, the slight increase in *R*_reten_ with declining hydraulic conductivity was also attributed to the poor retention effect of the whole bioretention system, which has already been mentioned above.

Recommendations for hydraulic conductivity of soil media vary from one country to another [2], with at least 12.5 mm/h in New Zealand and America, between 36 and 360 mm/h in Austria and between 50 and 200 mm/h in Australia. The saturated hydraulic conductivity of soils included in the soil catalog of the HYDRUS-1D model, taken from Carsel and Parrish [20], is shown in Table 4. Considering the conductivity requirement in different countries and its potential reduction with time, it is recommended that soils between loamy sand and sandy loam are used as the filter media in bioretention design enabling high hydrologic effectiveness (*R*_hydro_) and medium detention and retention effects (*R*_pr_, Δ*t*_dd_, Δ*t*_pd_, and *R*_reten_). Moreover, the ponding duration (*t*_pond_) in the simulations of loamy sand and sandy loam was shorter than 5 h, also maintaining better vegetation growth. In USEPA (1999), the sandy loam soil has already been recommended to be used in bioretention systems. In our experiment site, the saturated hydraulic conductivity of soil media in Cells A and B was, respectively, 49.6 and 151 mm/h (0.083 and 0.252 cm/min in Table 2), very close to the infiltration performance of sandy loam and loamy sand; thus, the two facilities both perform well in terms of water infiltration.

#### 3.2.4. Surface Areas

**Figure 8:**
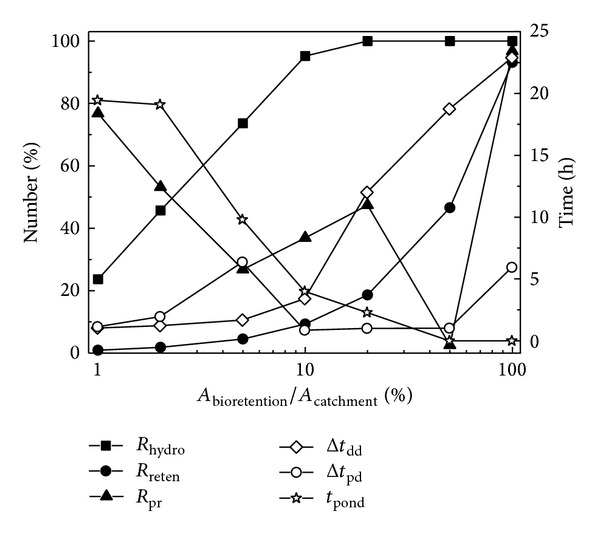
Hydrologic performance of bioretention measures under different bioretention areas of the catchment area.

General design guidelines suggest that the bioretention basin is approximately 5–7% of the effective upslope drainage area contributing to runoff (USEPA, 1999). With a surface area of 1–100% of the catchment area, the hydrologic performance of the bioretention is presented in Figure 8. As the surface area increased, the hydrologic effectiveness (*R*_hydro_) and water retention effect (*R*_reten_) both increased; meanwhile, the ponding duration (*t*_pond_) decreased. This agreed with the research results of Jones and Hunt [21] who suggested that large bioretention areas could reduce surface ponding times. However, the three hydrologic parameters for the water detention effect, bottom runoff delay (Δ*t*_dd_), bottom runoff peak flow delay (Δ*t*_pd_), and bottom runoff peak flow reduction (*R*_pr_), behaved differently with Δ*t*_dd_ increasing with the surface area, while Δ*t*_pd_ and *R*_pr_ changed drastically. This may be a function of the variation in hydrologic effectiveness, and, with a quick increase in *R*_hydro_ between area ratios of 1% and 10%, both Δ*t*_pd_ and *R*_pr_ changed irregularly. When *R*_hydro_ approached 100% with an area ratio larger than 10%, Δ*t*_pd_ increased gradually, but *R*_pr_ still had irregular variations, probably because of the combined effects of different inflow volumes and the constant hydraulic capacity of the filter media. However, it was evident that larger surface areas achieved better hydrologic performance, which was also confirmed by Le Coustumer et al. [2] who found that a larger surface area compensated for low conductivity by providing a greater filter area and ponding volume. From another perspective, Dussaillant et al. [22] showed that bioretention with an area of 10–20% of the contributing impervious area maximized groundwater recharge. However, in reality, as the bioretention area increases, the value of land increases especially in current Chinese cities and the facility becomes more costly. Considering the cost of land, a cost-effective surface area is recommended in bioretention design. From Figure 8, when the bioretention covers more than 10% of the catchment area, the total hydrologic effectiveness tends towards stability, and the water detention and retention effects change for the better; moreover, a ponding duration shorter than 4 h is also acceptable, and thus the surface area of 10% of the catchment area may be a reasonable compromise.

### 3.3. Long-Term Hydrologic Performance

**Figure 9:**
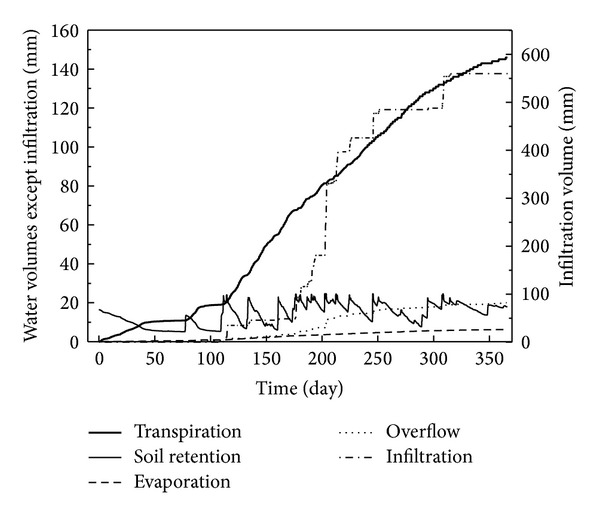
Accumulative water volumes variation with time in the long-term simulation using the annual meteorological data in 2012.

Using a medium filter depth of 60 cm, the filter type used in Cell A, which approximated the recommended sandy loam soil, and the recommended surface area of 10% of the catchment area, the long-term hydrologic performance of bioretention systems was assessed by inputting the annual meteorological data in 2012. The water volumes variation results are given in Figure 9. Because of the impervious sides and bottom surface of the bioretention system, it could be expected that the infiltration volume would take a large share of the total rainfall and the soil retention water may occupy a much lower percentage, which is proved in Figure 9 with the infiltration volume increasing rapidly with time, while the soil retention volume always fluctuates in the year. Meanwhile, the vegetation transpiration volume also increases evidently with time, which showed that, in the long run, evapotranspiration played an important role in the hydrology efficiency of bioretention systems. This was also confirmed by Dussaillant et al. [22], who reported that plant evapotranspiration during interstorm periods provided a greater available soil water storage capacity for the next rainfall event. It could be seen in Figure 9 that, after a year of operation, the infiltration, evaporation, transpiration, soil retention, and overflow volumes in the bioretention system were 560 mm, 6.3 mm, 146 mm, 1.4 mm, and 20 mm, respectively, contributing to 75.7%, 0.9%, 19.7%, 0.2%, and 2.7% of the total rainfall in 2012.

**Figure 10:**
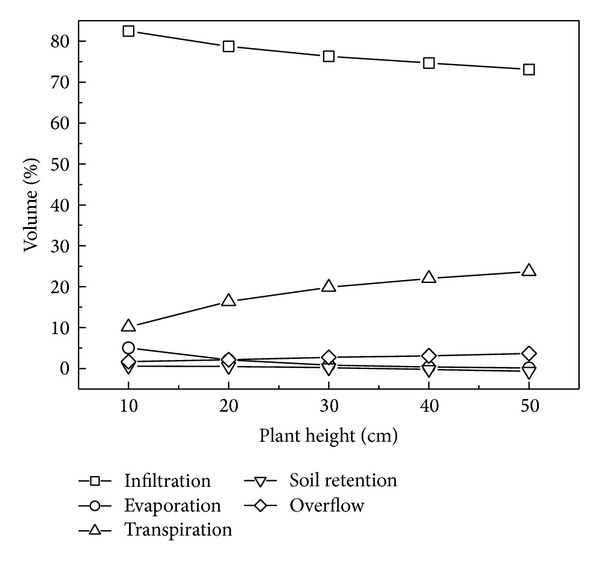
Water volume variation in bioretention systems with different plant heights.

Furthermore, some researchers provided that plants improved filter performance; for example, Archer et al. [23] reported that root growth increased hydraulic conductivity as a result of macropores created by root dieback, and Le Coustumer et al. [4] showed that plants with thick roots maintained system permeability over time compared with plants with finer roots. However, plants only influence evapotranspiration through their growth characteristics of height and root depth in HYDRUS-1D, and, as shown in Figure 10, evapotranspiration volume increased evidently with plant height. Presently, the model is unable to simulate the effect of plants on the permeability of the system.

## 4. Conclusions

Because stormwater management in urbanized areas has become ubiquitous, bioretention systems have been introduced as an effective source control technique to reduce runoff from impervious surfaces.

In this study, the hydrologic performance of two bioretention cells is modelled using HYDRUS-1D, with simulation results verified by field data. In the study, HYDRUS-1D accurately predicted infiltration and ponding processes in the bioretention cells.

The influence of different design parameters on the rainfall return period, media depth and type, and surface area to hydrologic performance was evaluated using the calibrated HYDRUS-1D model. It was shown that bioretention systems with underdrains and impervious boundaries have only some detention effect on bottom runoff delay, bottom runoff peak flow delay, and bottom runoff peak flow reduction, and their total water retention effect was very limited. Better detention effect was noted for smaller rainfall events, and a 2-year or less design storm was consequently recommended.

Filter media depth also had a significant impact on water detention but little or no effect on the total water treated. Better water detention appeared in deeper filter media, while, considering the filter cost, a cost-effective depth was recommended in bioretention design.

Both the hydraulic conductivity of filter media and surface area size influenced hydrologic effectiveness greatly, and better hydrologic effectiveness was reached with higher hydraulic conductivity and surface area ratio of the catchment area. Filter media with conductivity between loamy sand and sandy loam was recommended in bioretention design, enabling some conductive and retention effect as well as vegetation growth. Considering the cost of land, the cost-effective surface area was recommended in bioretention design, and the surface area of 10% of the catchment area may be a reasonable compromise.

Using the optimized design parameters for the rainfall return period, filter media depth and type, and surface area size, the long-term hydrologic performance of bioretention systems was further evaluated. As expected, the runoff inflow into the bioretention cell was mainly attenuated via infiltration, while at the same time evapotranspiration played an important role in the long run, contributing to 20.6% of the total rainfall in 2012.

Filter media play a very important role in hydrologic performance of bioretention measures, as conductivity and water retention capacity directly affect the infiltration, storage, and pollutant removal of inflow runoff. The pollutant transport process through the bioretention was not included in this study; thus, simulations of water quality improvement performance could be tried using the solute transport function of the HYDRUS-1D model, and, on this basis, the potential design parameters for better pollutant removal could be discussed, providing more references for the promotion and application of bioretention measures.

## Figures and Tables

**Table 1:** The input information in the model for Cells A and B.

Input information	Parameters	Values	
		Cell A	Cell B
Initial condition	Minimum water content	0.36	0.34
	Maximum water content	0.38	0.36
	Maximum height at soil (cm)	15	15

Geometry information	Number of layers	1	2
	Depth of the soil (cm)	60	25, 35

Time information	Time duration (min)	300	120
	Time step (min)	0.00001~5	0.001~0.25

Water flow-soil hydraulic property model	Model	van Genuchten-Mualem	

Water flow boundary conditions	Upper boundary condition	Atmospheric BC with surface layer	
	Lower boundary condition	Seepage face	

Vegetation properties	Water uptake reduction model	Feddes	
	Crop height (cm)	30	60
	Root depth (cm)	20	30

**Table 2:** The soil hydraulic parameters for the van Genuchten model in different structure layers.

Structural layer	Residual water content (cm^3^/cm^3^)	Saturation water content (cm^3^/cm^3^)	*α* (min^−1^)	*n* (—)	Saturated hydraulic conductivity (cm/min)	*l* (—)
Soil media in Cell A	0.065	0.410	0.025	1.69	0.083	0.5
Top media in Cell B	0.071	0.490	0.460	1.30	0.252	0.5
Bottom vermiculite in Cell B	0	0.448	0.002	1.44	0.518	0.5

**Table 3:** Statistics for simulation accuracy assessment.

Cell ID	RMSE		MRE		*R* ^2^	
	Artificial rainfall	Natural rainfall	Artificial rainfall	Natural rainfall	Artificial rainfall	Natural rainfall
A	0.014	0.003	0.16	0.22	0.95	0.76
B	0.036	0.017	0.23	0.25	0.97	0.61

**Table 4:** The saturated hydraulic conductivity of soils included in the soil catalog of the HYDRUS-1D model.

Soil type	Sand	Loamy sand	Sandy loam	Sandy clay loam	Loam	Silt loam	Clay loam	Silt	Clay
Saturated hydraulic conductivity (mm/h)	297	146	44	13.1	10.4	4.5	2.6	2.5	2
